# Are We Making the Most of Community Pharmacies? Implementation of Antimicrobial Stewardship Measures in Community Pharmacies: A Narrative Review

**DOI:** 10.3390/antibiotics10010063

**Published:** 2021-01-11

**Authors:** Doris Rusic, Josipa Bukić, Ana Seselja Perisin, Dario Leskur, Darko Modun, Ana Petric, Marino Vilovic, Josko Bozic

**Affiliations:** 1 Department of Pharmacy, University of Split School of Medicine, Soltanska 2, 21 000 Split, Croatia; doris.rusic@mefst.hr (D.R.); josipa.bukic@mefst.hr (J.B.); ana.seselja.perisin@mefst.hr (A.S.P.); dario.leskur@mefst.hr (D.L.); darko.modun@mefst.hr (D.M.); ana.petric@ljekarnasdz.hr (A.P.); 2 The Split-Dalmatia County Pharmacy, Dugopoljska 3, 21 204 Dugopolje, Croatia; 3 Department of Pathophysiology, University of Split School of Medicine, Soltanska 2, 21 000 Split, Croatia; marino.vilovic@mefst.hr

**Keywords:** antimicrobial stewardship, community pharmacy, antimicrobial resistance, respiratory tract infections, urinary tract infections, primary care

## Abstract

Community pharmacists recognize the need to implement antimicrobial stewardship activities in community pharmacies. They are in a unique position to provide triage for common primary care indications and to lower the burden of patients at general practitioners’ offices. However, research shows that, in some areas, dispensing of antimicrobials without valid prescription is still highly prevalent. Regardless of training, every community pharmacist can give his contribution to antimicrobial stewardship. One of the basic elements should be antimicrobial dispensing according to regulations, either prescription only, or according to guidelines where pharmacists have prescribing authority. Patient consultation supported with educational materials, such as leaflets, may reduce patients’ expectations to receive antibiotics for self-limiting infections and reduce pressure on general practitioners to prescribe antibiotics on patients’ demand. Treatment optimization may be achieved in collaboration with the prescribing general practitioners or by providing feedback. At last, pharmacists provided with additional training may be encouraged to provide consultation services to long-term care facilities, to introduce point-of-care testing for infectious diseases in their pharmacies or prescribe antimicrobials for uncomplicated infections. These services are welcomed by patients and communities. Expanding pharmacy services and pharmacists’ prescribing autonomy have shown a positive impact by reducing antibiotics consumption, thus ensuring better compliance with treatment guidelines.

## 1. Introduction

Antimicrobial resistance is among the leading global health threats to the modern world. Among the identified contributors to the emerging resistance is inappropriate antimicrobial consumption, which may be facilitated through increased antimicrobial availability in a community [1,2]. Preservation of public health requires every healthcare worker to be included in efforts to reduce the emergence of antimicrobial resistance [3]. With greater antibiotics consumption at outpatient settings, with estimates that around half of the antibiotics prescribed in the outpatient setting are unnecessary or inappropriate, there is an evident need for utilizing antimicrobial stewardship programs in outpatient settings [4]. According to Dyar et al., antimicrobial stewardship is “a coherent set of actions designed to use antimicrobials responsibly”. To combat rising antimicrobial resistance, several antimicrobial stewardship strategies have been proposed and implemented at the hospital and community level [3]. These include, but are not limited to, developing local treatment guidelines according to local pathogen-susceptibility data, establishing antimicrobial stewardship teams in tertiary care centers, preauthorization of prescriptions and post prescription audit and review, education and practical training, monitoring antimicrobial use and disease epidemiology [5,6].

Community pharmacists serve as gatekeepers to antimicrobial use. However, there are different regulations around the world; e.g., in some countries antimicrobials are prescription-only, in some there are topical antimicrobials available over the counter and in others most antimicrobials are sold without prescription. In Tanzania, dispensing of antibiotics without a prescription is common, although they are classified as prescription-only drugs. According to results of a study by Horumpende et al. antibiotics can easily be obtained without a prescription in up to 92% of pharmacies [7,8]. Even if designated as prescription-only, some pharmacists may dispense antimicrobials without a valid prescription and this issue seems to be more prominent in low- and middle-income countries, but is also widely present in some high-income countries [9,10,11,12,13,14,15,16,17,18,19]. A meta-analysis by Auta et al. on 38 studies in 24 countries estimated the overall proportion of antibiotics dispensed without a valid prescription at 62%. Only one of the included countries did not classify antibiotics as prescription-only drugs (Thailand). The proportion of dispensing non-prescribed antibiotics ranged from 8.2% for scenarios of symptoms of STDs and gastroenteritis in Zimbabwe to up to 97.6% in Saudi Arabia for patient requests for co-amoxiclav or cefaclor, indicating that antibiotics are frequently supplied without prescription, even in areas where this practice is illegal [18]. 

Ways in which to contribute to antimicrobial stewardship should be brought to the attention of every healthcare professional and stakeholder [3]. Publications describing community pharmacists’ efforts to contribute to reducing antibiotics consumption and describing community pharmacists’ participation in antimicrobial stewardship programs are included in this comprehensive review.

## 2. Antimicrobial Stewardship in Community Pharmacy

### 2.1. Awareness of and Barriers to Implementing Antimicrobial Stewardship Programs at the Primary Care Pharmacy Level

Pharmacists believe antimicrobial stewardship programs should be incorporated into community pharmacies, but that it requires them to receive more training and that any additional efforts should be adequately reimbursed [20,21,22,23,24]. Most pharmacists (more than 70%) believe that they play a key role in helping to control the use of antibiotics and consider educational campaigns as one of the most important strategies that can be adopted to combat antimicrobial resistance [25,26,27,28].

Pharmacists can discourage unnecessary visits to physicians and provide self-care advice when possible [29]. In addition to maintaining the drug supply in a community, they make efforts to prevent and reduce infections in the community, educate patients, communicate with prescribers, evaluate the appropriateness of the prescribed antibiotic and have a good understanding of the necessity of returning unused drugs for antimicrobial stewardship [30,31]. Some recent studies showed that there are still pharmacists that are unaware of antimicrobial stewardship programs, some have misconceptions about the development of antimicrobial resistance and some even report dispensing antimicrobials without a valid prescription [8,21,32,33]. 

A recent study investigating behaviors related to antimicrobial stewardship for respiratory tract infections in primary care found that 5 of 32 were community pharmacy behaviors, mostly patient education [34]. A study by Revolinski et al. showed varied antimicrobial stewardship practices in community pharmacies, with patient education and allergy assessment being among the most prevalent [35]. Even though pharmacists perceive the developed tools and guides as useful sources for patient education, they are poorly utilized among pharmacies due to poor dissemination [21,22,29]. There are other barriers in implementing different antimicrobial stewardship programs at the pharmacy level, including adequate funding, no prescribing options, no access to medical records and difficulties in contacting the consumers’ general practitioner [22,27,36].

Underutilization of pharmacists is possibly best described in a study by Charany et al., including 505 healthcare professionals from 53 countries, who revealed that the number of pharmacists who had postgraduate training in antibiotic management is less than for doctors and nurses (35% vs. 58% vs. 43%, respectively), but frequently reviewed prescriptions (90%) and developed guidelines more frequently then colleague doctors and nurses (57% vs. 47% vs. 18%); this indicates that pharmacists impose themselves as medication experts regardless of additional training [37].

### 2.2. Common Primary Care Indications

**Figure 1:**
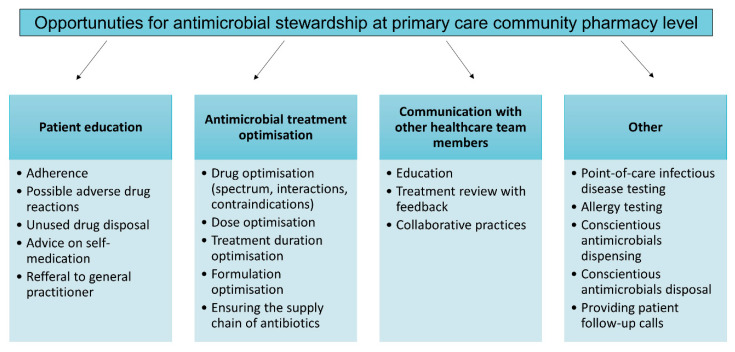
Antimicrobial stewardship opportunities in community pharmacies.

Community pharmacists remain the most accessible healthcare workers, and with most antimicrobials consumed at the primary care level, pharmacists are in a unique position to contribute to the management of antimicrobial resistance. With the estimated high accessibility of over-the-counter antimicrobials [18], pharmacists need to be reminded to rationally and conscientiously dispense antimicrobials. Opportunities for implementing antimicrobial stewardship at primary care community pharmacy level are summarized in Figure 1. 

Patient education and consultation on adequate self-medication when antibiotics are not needed may be the key element to handle patients who demand antimicrobials [38]. Even more, patient education should include stressing the importance of adherence to antimicrobial treatment and the importance of adequately disposing of unused drugs [31]. Some authors are problematizing the accordance of registered and available drug-pack sizes with treatment guidelines, further stressing the importance of patient adherence to the treatment. Even with perfect patient adherence to the treatment, there still might be leftover units of antibiotics that raise the risk of improper treatment in the future if these are left stocked at home [39,40,41]. Furthermore, community pharmacists play a key role in addressing patient concerns regarding possible adverse drug reactions, optimizing patient outcomes and referring patients to general practitioners when appropriate [42,43]. Other contributions of community pharmacists may include treatment optimization regarding dose, duration of the treatment or formulation where there is a well-established collaboration with the prescribers, alongside providing feedback to the prescribing physician and educating other healthcare team members [44]. Where possible, usually following additional training or in collaboration with a physician, community pharmacists may utilize immunization services, rapid infectious disease testing or even prescribe antimicrobials for common uncomplicated infections.

#### 2.2.1. Upper Respiratory Tract Infections

According to a study by Hersh et al., investigating the appropriateness of prescribed antibiotics for otitis media, sinusitis and pharyngitis (indications accounting for one third of prescribed antibiotics in outpatient setting), only 52% were found to be first-line agents [45]. Prescribers in outpatient settings are influenced by clinical uncertainty and time constrains and are often pressured by patients to prescribe antibiotics [46]. These observations highlight the need for outpatient antimicrobial stewardship [47].

Community pharmacists are recognized as antimicrobial stewards for upper respiratory tract infections, as they have the opportunity to communicate with both patients and prescribers and have specialist knowledge of drugs [47,48]. They can especially influence inappropriate antibiotics consumption, and among the possible targets for reducing inappropriate antibiotic consumption is a sore throat, as in most adult patients (around 90%) it is of viral origin [49].

Among the factors influencing the prescribers’ decision to provide antibiotics is diagnostic uncertainty [46]. There have been efforts to overcome this in a community pharmacy setting by providing “triage” and point-of-care testing to sore-throat patients and dispensing treatment in a community pharmacy where possible or referring to a physician when appropriate. The Centor score and modified Centor score are widely used to assess the etiology of pharyngitis using clinical presentations [50]. A study by Saengcharoen et al., conducted among more than 700 pharmacists in Thailand, evaluated how well pharmacists are diagnosing streptococcal pharyngitis and dispensing antibiotics. The results showed pharmacists’ knowledge on pharyngitis was negatively associated with dispensing antibiotics and pharmacists’ belief that patient satisfaction is achieved with receipt of antibiotics was associated with greater antibiotic dispensing. Moreover, pharmacists who were well familiar with the Centor criteria were taking more patient symptoms into account and were less likely to dispense antibiotics [51].

Such tools have been coupled with other scoring systems and further been expanded with point-of-care testing for infectious diseases [52,53]. Sensitivity and specificity estimates for these tests are promising, but cost-effectiveness of adopting them in a primary care setting is still debatable [54]. A group of researchers from France introduced point-of-care testing for detection of group A streptococci (GAS) across community pharmacies. Pharmacists participated in a 2 h training session on respiratory tract infection and training on rapid antigen-test use. The protocol consisted of a GAS pharyngitis risk assessment according to the modified Centor score and rapid antigen test for patients with a score of 2 or above. Patients were routinely instructed to consult a general practitioner if symptoms worsen or persist after 72 h. Based on the Centor score, GAS pharyngitis was suspected in 65.7% of patients, and 91.6% were further tested in pharmacies. These resulted in only 8.3% positive rapid antigen tests, accounting for 5% of the total included population. Patients who tested positive were instructed to consult a general practitioner since pharmacists in France do not have prescribing authority for antibiotics. Furthermore, all patients were provided with educational leaflets. Based on the follow-up on 38.5% of patients, all or almost all patients (99%) were satisfied with the rapid antigen test and positively judged the educational leaflets. Most pharmacists estimated the average time to conduct the entire protocol at 6 to 15 min, and 91.6% considered this duration appropriate and convenient. All the included pharmacists considered the rapid antigen test easy to use with 75.7% not having any difficulty performing the pharyngeal swab [52]. Upon introduction of a sore throat test and treating service across community pharmacies in Wales, pharmacists were trained on throat examination, scoring tools and sampling with a throat swab. A rapid antigen test was offered to patients scoring FeverPAIN > 3 or Centor > 2. After evaluation with the Centor or FeverPAIN scale, 72% of the patients were eligible for the rapid antigen diagnostics test, 28.2% tested positive and 27.4% received antibiotic treatment. For the purpose of the study, antibiotics could be supplied directly by the pharmacist under a Patient Group Direction. Follow-up was conducted on 51.9% of patients, of which 91.6% reported feeling better after using the service [55].

Introduction of a collaborative physician and community pharmacist GAS pharyngitis management program across community pharmacies proved that with physician consultation, pharmacists were able to identify patients likely to have GAS pharyngitis and provide timely treatments. Out of 86.4% patients eligible for testing, 17.6% had a positive test result. Follow-up (including 61.9% of patients) revealed that 76.3% of patients felt better, but 5.9% felt worse and 7.7% chose to seek additional care [56]. In another study offering GAS testing in community pharmacies, 48.8% of patients with a Centor score of 1 or 2 (not showing signs of a bacterial infection) would have consulted a general practitioner if the pharmacy service had not been available. Of those, 38.1% received a throat test, of which 59.5% tested negative. In total, antibiotics were supplied to 9.8% of patients directly by the pharmacist under the authority of a Patient Group Direction. The pharmacists’ completed training includes clinical examination, warning signs requiring referral and swabbing techniques. General practitioners’ offices located near the participating pharmacies were aware of the service and were encouraged to refer patients to the pharmacy. Considering the proportion of patients referred to general practitioners (*n* = 56) and patients who chose the pharmacy service instead of general practitioner consult (*n* = 97), possible savings were identified [57]. 

In another study, to overcome critics of the test accuracy, pharmacist offered influenza and GAS point-of-care tests based on rapid polymerase chain reaction according to a prespecified protocol using the Centor score scale; they performed the test, offered treatment with antibiotics, antivirals or over-the-counter drugs and followed-up with patients after 48 h. Among the patients tested for GAS, three-fourths reported feeling better, 13% were the same and just 1 patient felt worse. Among the patients tested for influenza, upon follow-up, two-thirds of the patients contacted reported feeling better, 17% reported feeling the same, and only 3.5% felt worse. Around 20% of patients were tested for both GAS and influenza, resulting in 4.7% positive GAS tests and 39.5% positive influenza tests. More than 25% of patients were seen on weekends or after 4 p.m. on weekdays, indicating such services offer greater accessibility to patients not only because of the wide community pharmacy distribution, but also because of convenient and longer working hours [58].

Research shows that antimicrobials are prescribed in 60% of cases of sore throat, hence there is space for reducing antibiotics prescribing, especially in this indication [59,60]. Other experience with point-of-care testing in pharmacy settings has shown that it may lead to reduced total antibiotics consumption, for example, drugs for antimalarial use [61].

Pharmacists gave positive feedback as to the feasibility of rapid antigen tests in pharmacies and considered the duration of the test acceptable, which is in direct contrast to general practitioners who identify a lack of time as the greatest barrier for rapid antigen test implementation in daily practice [52,62,63]. According to a published study, the average additional time of a point-of-care community pharmacy test for GAS was 25.3 ± 4.8 min, with an average pharmacist participation time of 12.7 ± 3.0 min. Total encounter time accounted for time from patient arrival, screening, patient completion of paperwork/review of symptoms, pharmacist consultation, physical assessment, performing the point-of-care test and waiting for the results and patient consultation on the treatment plan. Time to dispense a prescription, where appropriate, and time for recommendation of over-the-counter products for symptomatic treatment were not considered for total time, as both are part of the existing pharmacy workflow [64]. As described studies implicate, implementing sore throat test services in community pharmacies may reduce visits to a doctor’s office for uncomplicated sore throats [65]. Moreover, point-of-care testing promotes the choice of appropriate treatment and research shows that patients would be willing to use the service again even if they had to pay for such services [44,53,58]. 

In a study by Ashiru-Oredope et al., pharmacists underwent antimicrobial stewardship training and were encouraged to provide the Treat Antibiotics Responsibly, Guidance, Education, Tools: treating your infection—respiratory tract infection (TARGET TYI-RTI) leaflet to share with consumers who are seeking advice about self-limiting respiratory infections. The leaflet is both a resource and tool for clinicians to use during consultations and take-home information for patients, including information about the usual duration of common self-limiting respiratory infections, how to self-care and when to contact a healthcare professional. The study showed that providing community pharmacists with tools to enhance and optimize their interaction with patients was associated with a greater provision of self-care advice and less referral to the general practitioners for middle ear infections, sinusitis and coughs, especially for patients who received written information and were offered non-prescription drugs [25]. A study by van der Velden et al. further supports patient education in the treatment of sore throat, as the way healthcare providers communicate with patients can impact the treatment outcomes and patient satisfaction, stressing that patients seeking advice should be provided with written information about the management of sore throat [66]. 

In areas with a delayed antibiotic prescribing approach, pharmacists may facilitate its implementation by refusing to fill a delayed prescription (65%) or by providing additional advice to patients seeking antibiotics immediately after visiting general practitioners (29%) [67]. 

Actions that influence behavior in a way to enable responsible antimicrobial use or to limit inappropriate or unnecessary use of antimicrobials are key elements of antimicrobial stewardship [3]. Considering reports of many unnecessary or inappropriate antibiotics prescribed for sore throat, upper respiratory tract infections are a great opportunity for treatment optimization [68]. With most sore throat cases being of viral origin, good management of upper respiratory tract infections relies on patient education and on self-care and rapid diagnostics, both of which may be provided in a community pharmacy setting [49].

#### 2.2.2. Uncomplicated Urinary Tract Infections

Research shows that 25–30% of patients experiencing symptoms of urinary tract infection refer to a community pharmacy prior to going to a general practitioner. England uses the Treat Antibiotics Responsibly, Guidance, Education, Tools: urinary tract infection (TARGET UTI) leaflets designed to increase consumers’ confidence in self-care and to facilitate communication between healthcare professionals and consumers. Most patients were comfortable discussing symptoms if it was done privately and not over the counter. Pharmacists included in the study concluded that they could act as a first-line triage for consumers with urinary tract infections. Furthermore, the study identified the benefits of providing such advice in a community pharmacy setting rather than general practitioner’s office—no appointment needed, long working hours and pharmacists are educated and confident in providing advice. Pharmacist recognize that routinely providing patients with self-care advice for common uncomplicated infections is one of the key elements of their role, with the well-being and health of their patients being their motivation, rather than financial incentives. Moreover, ensuring patient compliance is the key responsibility for community pharmacists [36]. Time-saving resources are beneficial to assist pharmacists in providing self-care advice and treatment advice to patients. Providing patient education can not only prevent unnecessary visits to the general practitioner’s office and unnecessary antibiotics treatment, but can also prevent future antibiotics use, contributing to tackling antimicrobial resistance on multiple fronts [29]. 

Introducing pharmacist-led stewardship programs at a community-based, family medicine residency clinic, has proven to significantly contribute to the prescribing of appropriate antibiotics, doses and duration of treatment for uncomplicated cystitis and pyelonephritis [69].

Reclassification of trimethoprim in New Zealand gave pharmacists authority to treat some urinary tract infections following a specified training. Additionally, a screening tool is available to ensure the correct dispensing of the treatment. This practice did not appear to influence medical prescribing of antibiotics or to increase the overall use of antibiotics. Although only a small proportion of patients were eligible for pharmacist-prescribed trimethoprim, such practices may contribute to easing the burden and pressure to general practitioners’ offices and could also have a positive effect on reducing demands for antibiotic prescriptions from general practitioners [70]. In a study by Beahm et al., pharmacists performed patient assessments for symptoms of urinary tract infections, prescribed or modified treatment, provided education and referred to physicians when applicable. Follow-up was conducted after two weeks to evaluate adherence to treatment, adverse reactions and to confirm resolution of symptoms. Good adherence was established in more than 95% of patients and an initial cure was achieved in 94.5% of patients [71]. Research has confirmed the high confidence among pharmacists to provide treatment for uncomplicated urinary tract infections [72]. Such interventions showed significantly shorter time from decision to seek care from a pharmacist (1.7 ± 2.4 days) compared to time from decision to seek care from a physician (2.8 ± 3.8 days) [71]. This data is amplified with the great satisfaction of patients with pharmacists’ accessibility and a reduction in visiting a general practitioner’s office [71,73]. Furthermore, in patients who were prescribed drugs by a physician first, pharmacists modified 40.4% of prescriptions [71]. 

According to a study from Canada, management of uncomplicated urinary tract infections by pharmacist seems to also be of lower cost when compared to general practitioner and emergency physician-initiated management [74]. Additionally, such practices have shown that patients would be less likely to seek a general practitioner consultation if antibiotics became available through community pharmacies due to convenience [75].

### 2.3. Pharmacist as a Member of a Multidisciplinary Team

Pharmacists greatly contribute to the development and implementation of stewardship programs, they educate other health care members, and track, report and assess the effectiveness of stewardship strategies. Including a pharmacist is the starting point of antimicrobial stewardship program team building and can promote consensus in multidisciplinary healthcare teams regarding prescription rights [4,76,77]. In transition from inpatient to outpatient care, pharmacists may contribute to limiting overall inappropriate antimicrobial prescribing, optimize antibiotic selection, dose or reduce treatment duration [44].

Pharmacists that received additional training in infectious diseases and antimicrobial stewardship programs may assist in educating other members of the healthcare team and patients on elements of antimicrobial stewardship. Pharmacists can conduct antimicrobial regimen reviews and offer advice on doses and frequency optimization, as well as suggest discontinuation or change of treatment [35,44].

A study by Thornley et al., conducted among community pharmacists during pharmacist advice visits in long-term care facilities across England, found that pharmacists intervened for 9.5% of prescribed antibiotics. These interventions included clinical/allergy check (53.4%), issues with timing and continuation (32.2%), referral to a general practitioner (6.8%), error identified, sample recommended for testing and formulation change recommended (2.5% each) [78]. Such facilities have high rates of antibiotic consumption, offering community pharmacists a great opportunity to introduce infection prevention measures and ensuring the appropriate and effective use of antibiotics upon dispensing them [79].

In a study conducted in Japan in a retirement home with nursing care, pharmacists received 5-day training (workshops supervised by an infectious-disease specialist) on on-site Gram staining and assisted physicians on diagnosis and treatment selection. Sputum, urine and exudate samples were tested for pneumonia, urinary tract infections or cellulitis. If sampling could not be performed, pharmacist would advise on prescribing antibiotics that cover all likely pathogens. Researchers observed less prescribed antimicrobials per 100 residents with the total number of prescribed antimicrobials decreasing over 40% and continuing to drop over the course of the years. An increase in first-generation cephalosporin prescriptions was observed, alongside a decrease in third-generation cephalosporin prescriptions, indicating a wide- to narrow-spectrum switch post-intervention [80].

### 2.4. Other Considerations

#### 2.4.1. Penicillin Allergy

Beta-lactam antibiotics (penicillins, cephalosporins, carbapenems and monocyclic beta-lactams) make up to 65% of all injectable antibiotics in the United States [81]. Penicillins are among the most commonly dispensed antibiotics in primary care [82]. Therefore, it should come as no surprise that these antibiotics are widely present in treatment guidelines for common uncomplicated infections, especially respiratory tract infections of bacterial etiology [81]. Unfortunately, a reported penicillin allergy in patients may limit available treatment options for most common infections seen in primary care, such as such reactions may extend to other beta-lactams and discourage prescribers to administer them, pressuring second- and third-line antimicrobials for treatment. Other consequences of such a label include increased mortality risk during cancer and infection treatment, delay of initiation of appropriate antimicrobial therapy, increase in treatment failures or surgical infections, associated increase in multidrug-resistant infections and longer lengths of stay, all together leading to higher healthcare costs [83]. Penicillin allergy may be confirmed through evaluating the type of allergy/adverse drug reaction, interview and penicillin skin testing. Only a small proportion of patients with reported penicillin allergy have a history of a high-risk reaction. Numerous studies suggest that the proportion of negative allergy penicillin skin tests in patients with documented penicillin allergies ranges from 79% to 100% [83,84,85]. Such services are usually pharmacist-directed under a collaborative practice agreement [86,87]. Furthermore, these services can be implemented in an outpatient setting with pharmacists doing an in-depth interview and referring eligible patients for further testing or suggesting immediate allergy de-labelling based on the gathered information [88]. Although there are examples of utilization of penicillin allergy tests in outpatient settings, this service cannot be fully implemented in a community pharmacy due to related risks (i.e., probability of anaphylaxis) [89]. However, community pharmacists can conduct detailed interviews that can offer the basis for de-labelling and refer eligible patients to testing [90]. Removal of an unnecessary label is cost-effective and avoids bad treatment outcomes, treatment failures, surgical infections and multidrug-resistant infections [83]. 

#### 2.4.2. Veterinary Drugs

Another field of interest concerns veterinary medicine, as community pharmacists are increasingly being involved in dispensing animals’ medications [91,92]. The extensive use of antibiotics in animal husbandry is considered an important contributor to microbial resistance, as antibiotics are not only used in treatment of developed infection but also as a growth promotor factor or prophylactic, often administered to an entire stock of animals [93,94]. Manure and other animal waste, as well as wastewater from farms, could be contaminated with antibiotics, their metabolites and resistant strains of bacteria [95,96,97]. Their improper disposal could also contaminate food sources, further propagating their resistance [96,98,99]. A survey found that roughly half of Italian veterinarians working on pig and cattle farms considered antimicrobial usage as not always in line with the guidelines [100]. In developing countries such as Ghana, more than 80% of farmers admitted they had bought antimicrobials over-the-counter without a prescription. The same study found that only 8.5% of antimicrobials were administered by veterinarians while the rest were administered by the farmers themselves, mostly based on their own experience rather than advice from healthcare workers [97]. In certain cases, the antibiotic was used in the form of an active pharmaceutical ingredient, instead of a finished drug product, and was usually added to animals’ feed [101]. Moreover, some antibiotics used were the ones meant for human use and considered critical for human health [102,103].

Antibiotic misuse in pet animals also presents risks due to longer contact with their owners, which could enable the transmission of resistant strains of bacteria to humans [93]. The World Organization for Animal Health recognized the potential contribution from the pharmacists in their guidelines for the responsible use of antibiotics in veterinary medicines. The guidelines propose the appropriate dispensing, labelling and record-keeping of the dispensed antimicrobials and other responsibilities of the pharmacists [104]. Despite their efforts, there were still cases where antimicrobial medications had been dispensed without valid prescription, which was especially prevalent in low- and middle-income countries, where the prescribing regulations were not adequately enforced [102,105]. In such countries, a pharmacist could have a more prominent role in antimicrobial stewardship. However, they seemed to be underutilized, as the farmers in those countries did not seek their advice about antibiotic choice, dosage or use [97,103]. Especially useful could be the pharmacists’ role as educators, as farmers are often aware of their overuse of antibiotics, or the existence of antimicrobial resistance, but still continue with their practice as they do not know its consequences on public health or alternative methods to prevent diseases [96,98,103,106]. Another concerning practice was discovered by a study in India where the veterinary antimicrobial medications for working animals were dispensed intentionally in wrong dosages and in higher quantities for financial gain [105]. Another obstacle in ensuring the proper use of antimicrobial drugs was the pharmacists’ perceived lack of knowledge about veterinary pharmacology, further potentiated by the lack of developed guidelines about antibiotics use in animals [91,92].

## 3. Conclusions

Community pharmacists recognize the need of implementing antimicrobial stewardship activities in community pharmacies. They are in a unique position to provide triage for common primary care indications and to lower the burden of patients to general practitioners’ offices. One of the basic elements should be antimicrobial dispensing according to regulations, either prescription-only, or according to guidelines where pharmacists have limited prescribing authority. This should be accompanied with patient consultation that may be facilitated using indication-specific education material (i.e., leaflets), which has proved to facilitate pharmacists–patient communication. Patients’ concerns should be addressed about possible adverse drug reactions, treatment duration, general practitioner referral and drug disposal. Patient education further may reduce patients’ expectations to receive antibiotics for self-limiting infections and reduce pressure on general practitioners to prescribe antibiotics on patients’ demand. Treatment optimization may be achieved through dose, duration of treatment and formulation optimization, either in collaboration with the prescribing general practitioners or by providing feedback. However, these are dependent on the general practitioners’ willingness to participate in such programs. At last, community pharmacists provided with additional training may be encouraged to provide consultation services to long-term care facilities, to introduce rapid antigen testing and point-of-care testing for infectious diseases and respiratory tract infections in their pharmacies, or prescribe antimicrobials for uncomplicated infections. These services are welcomed by patients and communities. Expanding pharmacy services and pharmacists’ prescribing autonomy have shown positive impacts by reducing antibiotics consumption, thus ensuring compliance with treatment guidelines.

## Data Availability

Not applicable.
